# An artificial intelligence-based approach for identifying the proteins regulating liquid–liquid phase separation

**DOI:** 10.1093/bib/bbaf313

**Published:** 2025-07-09

**Authors:** Zahoor Ahmed, Kiran Shahzadi, Rui Li, Yu-Qing Jiang, Yan-Ting Jin, Muhammad Arif, Juan Feng

**Affiliations:** The Clinical Hospital of Chengdu Brain Science Institute, School of Life Science and Technology, University of Electronic Science and Technology of China, Chengdu, 611731 Sichuan, China; School of Medical Technology and Information Engineering, Zhejiang Chinese Medical University, Hangzhou, 310053 Zhejiang, China; Department of Biotechnology, Women University of Azad Jammu and Kashmir Bagh, Bagh, Azad Kashmir 12500, Pakistan; The Clinical Hospital of Chengdu Brain Science Institute, School of Life Science and Technology, University of Electronic Science and Technology of China, Chengdu, 611731 Sichuan, China; The Clinical Hospital of Chengdu Brain Science Institute, School of Life Science and Technology, University of Electronic Science and Technology of China, Chengdu, 611731 Sichuan, China; The Clinical Hospital of Chengdu Brain Science Institute, School of Life Science and Technology, University of Electronic Science and Technology of China, Chengdu, 611731 Sichuan, China; College of Science and Engineering, Hamad Bin Khalifa University, Doha 34110, Qatar; The Clinical Hospital of Chengdu Brain Science Institute, School of Life Science and Technology, University of Electronic Science and Technology of China, Chengdu, 611731 Sichuan, China

**Keywords:** LLPS, regulator proteins in LLPS, ESM2_t36, multilayer perceptron

## Abstract

Liquid–liquid phase separation (LLPS) is a biomolecular process that underpins the formation of membrane-less organelles within living cells. This phenomenon, along with the resulting condensate bodies, is increasingly recognized for its critical roles in various biological processes, such as ribonucleic acid (RNA) metabolism, chromatin rearrangement, and signal transduction. Notably, regulator proteins play a central role in the process of LLPS. They are essential for the formation, stabilization, and maintenance of the dynamic properties of LLPS, ensuring an appropriate phase separation response to cellular signals. Targeting these regulator proteins is the key to manipulating LLPS for applications in biotechnology, materials science, and medicine, including biomaterials, drug delivery, diagnostics, and synthetic biology. Given their importance, this study focused on an artificial intelligence-based approach to identify regulator proteins in LLPS. We constructed a dataset of 913 positive and 6584 negative protein sequences, and divided it into eight balanced training datasets and a test dataset. Semantic information from protein sequences was extracted using the ESM2_t36 pretrained protein language model, followed by training a multilayer perceptron classifier. The model achieved 0.78 accuracy on the test dataset, outperforming traditional sequence-based methods, one-hot encoding, and other pretrained embedding methods. SHapley Additive exPlanations (SHAP)-based interpretation revealed key biophysical patterns enriched in regulator proteins, including higher levels of charged and disordered residues. Our results show that deep contextual protein representations combined with neural network-based classifiers can accurately identify LLPS regulator proteins. This tool offers new opportunities for understanding condensate biology and designing synthetic phase-separating systems. All data and code are available at: https://github.com/bioplusAI/LLPS_regulators_pred.

## Graphical Abstract

**Graphical Abstract:**
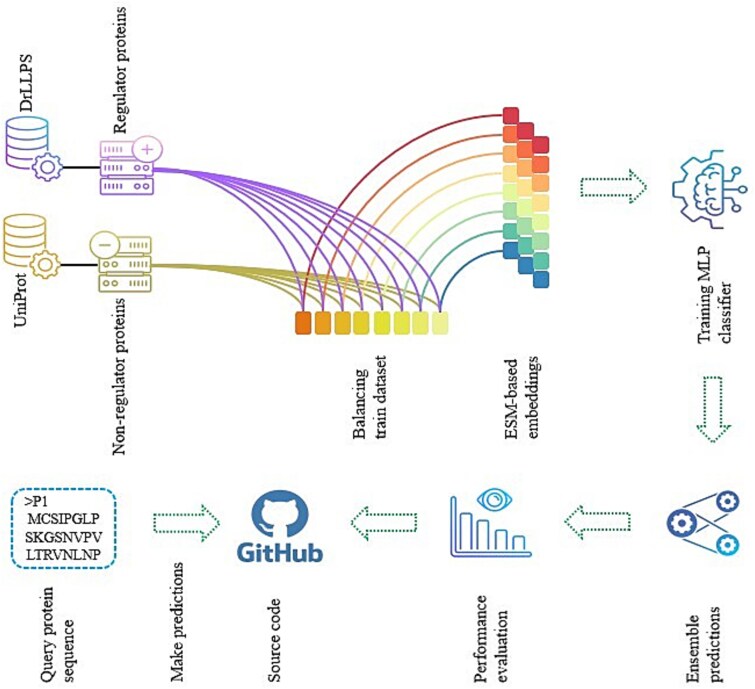

## Introduction

Liquid–liquid phase separation (LLPS) refers to the process by which a homogenous solution of biomolecules, such as proteins or nucleic acids, separates into two distinct liquid phases [1]. This phenomenon is observed in cellular biology and plays a critical role in organizing biochemical reactions within the cell [2–5]. LLPS is essential for the formation of membrane-less organelles (MLOs) such as nucleoli, stress granules, and P-bodies, which compartmentalize and regulate various cellular processes [6, 7]. LLPS is mainly driven by two types of proteins: scaffolds and clients. Scaffold proteins derive the formation of the LLPS by interacting with other scaffolds and client proteins. In contrast, client proteins are not directly involved in the phase separation but instead integrate into pre-existing phase-separated droplets and undergo concentration-dependent changes, potentially modifying their function or activity. These proteins often perform a functional role in the phase-separated environment, such as facilitating biochemical reactions [8]. In addition to scaffolds and clients, several proteins associated with MLO scaffolds have been identified as key regulators of LLPS stability and formation. These proteins are referred to as regulator proteins. Regulator proteins influence the LLPS process by modulating the interactions among phase-separating proteins. They can enhance or suppress LLPS by modifying post-translational processes, such as phosphorylation, or by altering the surrounding environment, including ionic strength or temperature [9–11].

In the study of LLPS, regulator proteins are critical in controlling phase behavior, making them the central to the design of synthetic LLPS systems with tailored properties. By engineering specific regulator proteins, it is possible to create functional LLPS constructs with desired characteristics, such as stability, responsiveness to stimuli, and targeted release. Modifying amino acid sequences or post-translational modifications of these proteins can further refine interactions within LLPS systems, enabling precise responses to environmental stimuli [12, 13]. The potential applications of synthetic LLPS in biotechnology are extensive. In drug delivery, e.g. regulator proteins can help design systems that release therapeutics in response to specific cellular signals, improving treatment efficacy [14–18]. Additionally, synthetic LLPS in cell-free systems can facilitate the formation of biochemical pathways, offering controlled environments to study cellular processes [19, 20]. Therapeutically, targeting regulator proteins is promising for diseases associated with LLPS dysregulation, such as neurodegenerative disorders, allowing the development of strategies to restore normal phase separation dynamics [21, 22]. Furthermore, in synthetic biology, regulator proteins can enable precise control over gene regulation by creating conditions that trigger specific LLPS events, thus opening new avenues for innovative applications in gene expression and cellular function [12, 18, 23–25]. This underscores regulator proteins’ importance in natural and synthetic LLPS systems and their potential to drive advances across multiple fields.

Despite the biological importance of LLPS regulators, computational studies have largely focused on scaffold or client proteins, with relatively few efforts directed toward the systematic identification of LLPS regulator proteins. This gap is partly due to limited annotation data and the lack of distinct sequence features. However, recent advances in protein language models offer new opportunities for capturing hidden semantic and evolutionary information from raw sequences, providing a potential pathway for high-throughput prediction.

In this study, we propose a novel artificial intelligence-based framework to identify LLPS regulator proteins directly from sequence data. We first constructed a comprehensive dataset consisting of 913 positive and 6584 negative protein sequences. To construct the test dataset, 113 positive and 184 negative sequences were randomly selected, resulting in a final test dataset of 297 sequences. The remaining 800 positive sequences were retained for training purposes. To address the issue of class imbalance, the remaining 6400 negative sequences were randomly partitioned into eight equal subsets. Each subset was then combined with the positive training set to generate eight balanced training datasets, ensuring equal representation of positive and negative samples in each. Then, we extracted feature information from these protein sequences using ESM2_t36, a pretrained protein language model. Subsequently, we trained an ensemble predictor based on a multilayer perceptron (MLP) classifier, which achieved an accuracy of 0.78 on the test dataset through 10-fold cross-validation. We compared our ESM2_t36-based method with traditional sequence-based feature approaches, one-hot (1H) encoding, and other pretrained protein language models. Our method consistently outperformed these alternatives, underscoring the effectiveness of leveraging the powerful ESM2_t36 model in combination with class balancing strategies and advanced deep learning techniques for the accurate identification of regulator proteins associated with LLPS. This work represents one of the first efforts to computationally predict LLPS regulator proteins and provides a scalable tool to support both basic research and synthetic applications involving biomolecular condensates.

## Materials and methods

**Figure 1:**
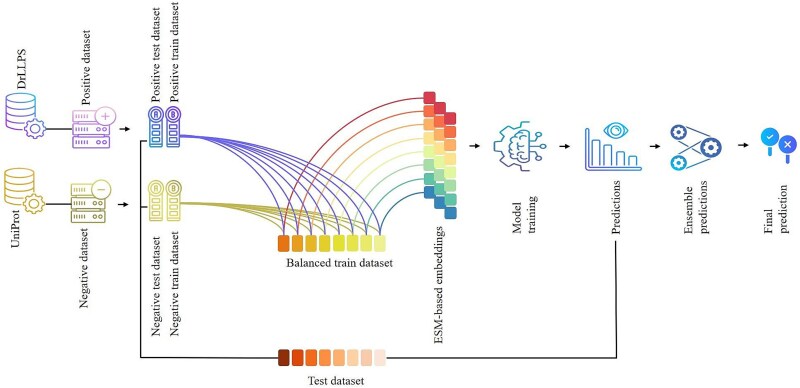
Flow chart of the framework of the present study.

Figure 1 presents the flow chart of the present study. The study includes (i) benchmark dataset construction, (ii) class balancing (iii) feature representation, (iv) model training, and (v) validation.

### Dataset

**Figure 2:**
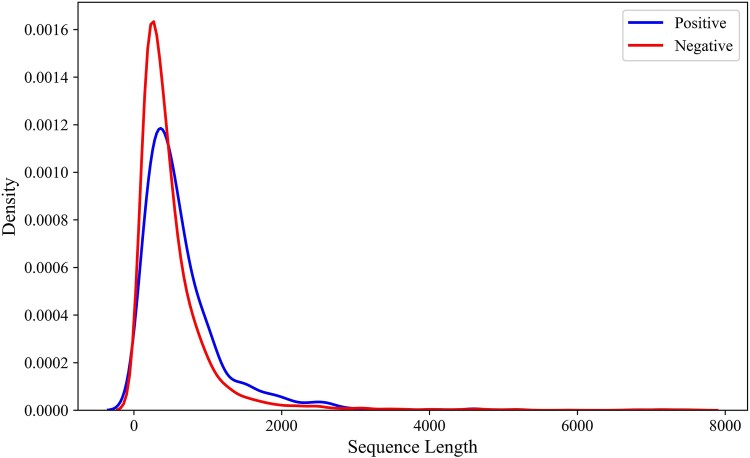
Distribution of sequence lengths in the positive and negative sequences.

A reliable dataset is the foundation of a high-performance prediction model [26–31]. Considering that, we assembled a positive dataset of 987 experimentally verified regulator proteins involved in LLPS, sourced from the DrLLPS database [32]. For the negative dataset, we extracted protein sequences from UniProt [33], based on the assumption that they are unlikely to play a regulator role in LLPS, including cytoskeletal proteins, membrane-associated proteins, highly structured proteins such as globular proteins, proteins involved in forming stable structures, and single-domain proteins. To eliminate redundant sequences that could increase computational load and timing, we used the CD-HIT tool [34] with a cut-off value of 70% for the positive dataset and 40% for the negative dataset. After this curation process, we obtained 913 positive and 6584 negative protein sequences. To ensure balanced training datasets, we randomly selected 113 positive sequences from the positive dataset for the test set, leaving 800 positive sequences for training. Similarly, 184 negative sequences were randomly selected for the test set, leaving 6400 negative sequences for training. These remaining negative sequences were randomly divided into eight subsets, each containing 800 sequences to match the 800 positive sequences and ensure perfectly balanced training datasets. As a result, we obtained eight balanced training datasets. The selected positive and negative test sequences were then combined, resulting in a test dataset containing 297 protein sequences. By prioritizing a balanced training dataset, we ended up with a slightly higher proportion of negative sequences in the test dataset. The majority of the positive and negative sequences were shorter than 2000 amino acids (Fig. 2). Furthermore, the positive sequences exhibited lower hydrophobic, more hydrophilic, more positively and negatively charged residues (Table 1) as compared to negative sequences, which is consistent with the expected biophysical behavior of proteins regulating LLPS.

**Table 1:** Distribution of hydrophobic, hydrophilic, and charged residues in positive and negative sequences

**Physicochemical properties**	**Positive sequences**	**Negative sequences**
Hydrophobic residues	0.37	0.39
Hydrophilic residues	0.50	0.48
Positively Charge residues	0.15	0.14
Negatively Charge residues	0.13	0.12

### Feature representation

We employed three categories of feature representation methods: (i) traditional sequence-based features, (ii) 1H encoding, and (iii) embeddings from pretrained protein language models.

### Traditional sequence-based feature embeddings

The sequence-based features, AAC (amino acid composition), DPC (dipeptide composition), TPC (tripeptide composition), and CTD (composition, transition, distribution), were employed as embeddings for protein sequences in training models to predict proteins regulating LLPS. These features represent distinct aspects of a protein sequence: AAC provides an overview of the amino acid distribution, DPC and TPC capture local and global interactions between adjacent amino acids, and CTD highlights transitions in AAC, which may help identify disordered regions that are critical for protein regulating LLPS. For the generation of these sequence-based feature embeddings, we used the iFeature tool [35].

#### Amino acid composition

AAC refers to the frequency of occurrence of each of the 20 standard amino acids within a given protein sequence. It is calculated using the formula:


(1) \begin{equation*} f(m)=\frac{N(m)}{N} \end{equation*}

where *f(m)* denotes the frequency of amino acid *m*, *N(m)* is the number of occurrences of amino acid *m* in the protein sequence, and *N* is the total number of amino acids in the sequence [36].

#### Dipeptide composition

DPC quantifies the frequency of occurrence of all possible dipeptides within a protein sequence. It is calculated using the formula:


(2) \begin{equation*} Dc(AC)=\frac{N(AC)}{N-1} \end{equation*}

where *Dc(AC)* represents the frequency of the dipeptide consisting of amino acids *A* and *C*, *N(AC)* is the number of times this specific dipeptide appears in the sequence, and *N−1* is the total number of dipeptides in the protein [37].

#### Tripeptide composition

TPC measures the frequency of occurrence of all possible tripeptide sequences of three consecutive amino acids within a protein sequence. It is defined by the formula:


(3) \begin{equation*} Tc(ACD)=\frac{N(ACD)}{N-2} \end{equation*}

where *Tc(ACD)* denotes the frequency of the tripeptide consisting of amino acids *A*, *C*, and *D*; *N(ACD)* is the number of times this specific tripeptide appears in the sequence; and *N−2* is the total number of tripeptides in the protein [36].

#### Composition, transition, distribution

The 20 standard amino acids can be classified into three main categories polar, neutral, and hydrophobic based on their physicochemical properties. The CTD descriptor is designed to capture both overall and position-specific characteristics of these amino acid groups within a protein sequence. The Composition (C) component represents the relative frequency of residues belonging to each group across the entire sequence. The Transition (T) component measures how often residues from one group are immediately followed by residues from a different group. The Distribution (D) component identifies the sequence positions where the first 25%, 50%, 75%, and 100% of the residues from each group occur. The mathematical formulation of the composition feature is given as:


(4) \begin{equation*} Cr=\frac{Nr}{N} \end{equation*}

where *N(r)* denotes the number of residues of type *r*, *r* can be polar, neutral, hydrophobic, and *N* is the total length of the protein sequence [38].

For detailed information about these traditional sequence-based features refer to reference [35].

### One-hot encoding

1H encoding is a commonly used technique for representing amino acid sequences in numerical format. 1H encoding represents each amino acid as a binary vector of length 20, where only one element is 1, indicating the presence of that amino acid, and all others are 0.


(5) \begin{equation*} {v}_i\left[j\right]=\left\{\begin{array}{@{}ll}1, & if\ j=\left({a}_i\right)\\{}0, & otherwise\end{array}\right. \end{equation*}

where *j* ranges 1 to 20, and index (${a}_i$) maps the amino acid to a unique position in the vector [39].

### Pretrained protein language model

In addition to traditional sequence-based features and 1H encoding, we also utilized protein pretrained language models (PLMs), including evolutionary scale modeling2 (ESM2), Universal representation (UniRep), and ProtT5, to generate dense contextualized embeddings.

#### Evolutionary scale modeling2

ESM2 is a transformer-based protein language model that applies natural language processing techniques to protein sequences, treating them as a biological language. Pretrained on an extensive protein dataset, ESM2 excels at identifying patterns related to protein evolution and function [40]. Compared to traditional models, ESM2 leverages a powerful transformer architecture capable of capturing long-range dependencies within sequences, providing superior generalization for tasks such as protein structure and function prediction [41]. The embeddings generated by ESM2_t36 have a shape of (L, D), where L represents the sequence length, and D represents the embedding dimension, typically ranging from 768 to 2560. To address the issue of variable sequence lengths, we set the maximum sequence length to match that of the longest protein sequence in the dataset. Sequences shorter than this length were padded accordingly to ensure consistent input dimensions across all samples. These embeddings capture critical structural and functional information, making them valuable for downstream tasks such as protein classification [42].

#### Universal representations

UniRep is a deep learning-based pretrained model developed to generate fixed-length vector embeddings from protein sequences. It employs a recurrent neural network architecture, specifically utilizing long short-term memory units, to model the sequential nature of amino acid chains. By training on large-scale protein sequence datasets, UniRep captures underlying biological patterns, encoding features that reflect both evolutionary relationships and structural characteristics. The resulting representations provide a compact and informative summary of protein properties, making them valuable for various bioinformatics applications, including protein function prediction, classification tasks, and folding simulations [43].

#### Prot5

ProtT5 is a transformer-based protein language model adapted from the T5 architecture, which was initially designed for natural language processing tasks. Leveraging large-scale protein sequence datasets such as Big Fantastic Database and UniRef50, ProtT5 is trained to generate contextualized representations of amino acid sequences without the need for explicit structural data. By encoding each residue in the context of its neighboring residues, the model effectively captures evolutionary, structural, and functional patterns. These rich embeddings serve as informative input features for a variety of downstream applications, including protein classification [44].

### Model training

The extracted protein feature representations for each balanced training dataset were used as input features for model training [45, 46]. Four classifiers, support vector machine (SVM), XG-boost (XGB), multilayer perceptron (MLP), and convolutional neural network (CNN), were used to train the models, with 10-fold cross-validation applied to each balanced training dataset to ensure robust training and validation. Given the simplicity and efficiency, SVM was utilized to train a model based on traditional sequence-derived features. During the training process, custom callbacks were employed to monitor validation accuracy and retain the best-performing model weights for testing. This process resulted in the training of eight individual models. To combine the predictions from these models, an ensemble prediction was performed using majority voting of the outputs. In cases of a tie between positive and negative votes, the final decision was made by calculating the average probability and applying a threshold of 0.5. Finally, the ensemble approach was validated on the test dataset.

### Support vector machine

SVM projects input features into a high-dimensional space and identifies an optimal hyperplane that maximizes the separation between two classes, utilizing a suitable kernel function. Various kernel functions can be employed depending on the nature of the data. Given the nonlinear characteristics of our dataset, the radial basis function (RBF) kernel was selected. The RBF kernel between two vectors *m* and *n* is defined as:


(6) \begin{equation*} K\left(m,n\right)=\exp \left(-\gamma \parallel m-n{\parallel}^2\right) \end{equation*}

where $K\left(m,n\right)$ is a kernel function measuring the similarity between two vectors *m* and *n*, and $\gamma$ is a tunable parameter that influences the decision boundary’s flexibility. For a comprehensive explanation of SVMs, refer to [47].

### XG-boost

XGB employs gradient-boosted decision trees for predictive modeling, where the final prediction is obtained as the summation of predictions from all individual trees [48–50]. It optimizes the objective function, which combines a loss function *l* and a regularization term $\Omega$, to prevent overfitting and improve generalization. The general form of the objective function can be expressed as:


(7) \begin{equation*} obj=\sum_{i=1}^nl\left({y}_t,{y}_p\right)+\sum_{k=1}^K\Omega ({f}_k), \end{equation*}

where $n$ represents the number of samples, *yt*, and *yp* correspond to the true and predicted values for the data sample *i*, respectively. The term $l\left({y}_t,{y}_p\right)$ donates the loss function, *K* and *fk* represent the number of the trees and the *k-th* tree, respectively. $\Omega \left({f}_k\right)$ is a regularization term that helps to mitigate overfitting.

### Multilayer perceptron

MLP classifier is a feedforward neural network commonly used for supervised tasks like classification and regression [51–53]. It comprises an input layer, several hidden layers, and an output layer, with each neuron fully connected to those in neighboring layers. Mathematically, MLP can be expressed as:


(8) \begin{equation*} {y}_p=g\left({W}^{(l)}.f\left({W}^{\left(l-1\right)}.f\left(\dots \kern0.5em .f\left({W}^{(1)}.x+{b}^{(1)}\right)\dots \right)+{b}^{\left(l-1\right)}\right)+{b}^{(l)}\right) \end{equation*}

where, $x$ represents input data, ${w}^l$ weight matrix represents *l-th* lawyer, ${b}^l$ is the biased function of the *l*-*th* layer, ${f}^l$ is the activation function of *l*-*th* layer, $g$ represents the activation function of the output layer, while ${y}_p$ is output.

In this study, MLP architecture included an input layer with 2560 neurons, followed by hidden layers containing 128, 64, 32, and 16 neurons, each utilizing the ReLU activation function. The output layer, designed for binary classification, consisted of a single neuron with a sigmoid activation function. Training this MLP involved backpropagation to reduce a binary cross-entropy loss, using the Adam optimizer with a learning rate of 0.001.

### Convolutional neural network

CNN is a type of deep learning neural network architecture known for its effectiveness in capturing spatial hierarchies of features from protein data [54–57]. CNN architecture consists of layers that use convolution filters to capture patterns from input data. In this research context, the CNN consisted of two 1D convolution layers, and four fully connected layers were used, as depicted by the following equation.


(9) \begin{equation*} \left(I\ast K\right)\left(i,j\right)=\sum m\sum n\left(i+m,j+n\right).K\left(m,n\right) \end{equation*}

where *I* is the input, *K* is the convolutional kernel, and * denotes the convolution operation. $\left(i,j\right)$ represents the spatial position in output, and $\left(m,n\right)$ iteration over spatial dimensions. Pooling layers then reduce the spatial dimensions of the feature maps, minimizing computational load and mitigating overfitting. The final fully connected layers aggregate the learned features for classification tasks [58].

### Computational cost and hardware requirements

All models were trained and evaluated on an NVIDIA RTX 3060 Ti GPU (8GB VRAM). The ESM2_t36 model required ~3.62 s per sequence for embedding generation. Full training time per dataset was around 1.6 h, and prediction time per test sequence was ~4 s.

### Performance evaluation

To assess the model’s performance, we utilized metrics such as sensitivity *(SP)*, specificity (*SP*), accuracy (*ACC*), the area under the curve (*AUC*), *F1*, and Matthew’s correlation coefficient (*MCC*) [59–61]. *SN* measures the model’s ability to correctly identify regulator proteins in LLPS, while *SP* evaluates its accuracy in distinguishing non-regulator proteins. ACC offers an overall evaluation of the model’s performance across both regulator and non-regulator classes. *AUC*, derived from the Receiver Operating Characteristic (*ROC*) curve [62–64], is particularly significant in binary classification tasks [65, 66], as it reflects the model’s capacity to separate positive and negative classes. The *F1* represents the harmonic mean of precision and recall, and is particularly useful for evaluating model performance in scenarios with imbalanced datasets. While, *MCC* serves as a comprehensive measure of binary classification quality, considering all elements of the confusion matrix. The mathematical formulations for these metrics are detailed below:


(10) \begin{equation*} SN=\frac{TP}{TP+ FN} \end{equation*}

(11) \begin{equation*} SP=\frac{TN}{TN+ FP} \end{equation*}

(12) \begin{equation*} ACC=\frac{TP+ TN}{TP+ FP+ TN+ FN} \end{equation*}

(13) \begin{equation*} AUC={\sum}_{i=1}^{n-1}\frac{\left({m}_{i+1}-{m}_i\right).\left({n}_i-{n}_{i+1}\right)}{2} \end{equation*}

(14) \begin{equation*} F1=\frac{2\times Precision\times Recall}{Precision+ Recall} \end{equation*}

(15) \begin{equation*} MCC=\frac{TP\times TN- FP\times FN}{\sqrt{\left( TP+ FN\right)\left( TN+ FN\right)\left( TP+ FP\right)\left( TN+ FP\right)}} \end{equation*}

here, *TP* signifies true positives, where regulator proteins are accurately identified as regulator proteins. *TN* denotes true negatives, representing instances where non-regulator proteins are correctly identified as non-regulator proteins. *FP* denotes false positives, where non-regulator proteins are erroneously categorized as regulator proteins, while *FN* represents false negatives, indicating instances where regulator proteins are incorrectly labeled as non-regulator proteins [67–70]. In the context of *AUC*, ${m}_i$, and ${n}_i$ represent the true positive rate and false positive rate, respectively.

## Results and discussion

### Model evaluation

#### Performance evaluation of multilayer perceptron-based ESM2_t36 model

To systematically evaluate the effectiveness of the MLP-based ESM2_t36 model in identifying LLPS regulator proteins, we conducted 10-fold cross-validation on eight balanced training datasets and performed independent testing on a hold-out test dataset. Each training dataset was carefully constructed to maintain a 1:1 ratio of positive and negative samples, ensuring consistent learning conditions and reducing potential bias from class imbalance.

**Figure 3:**
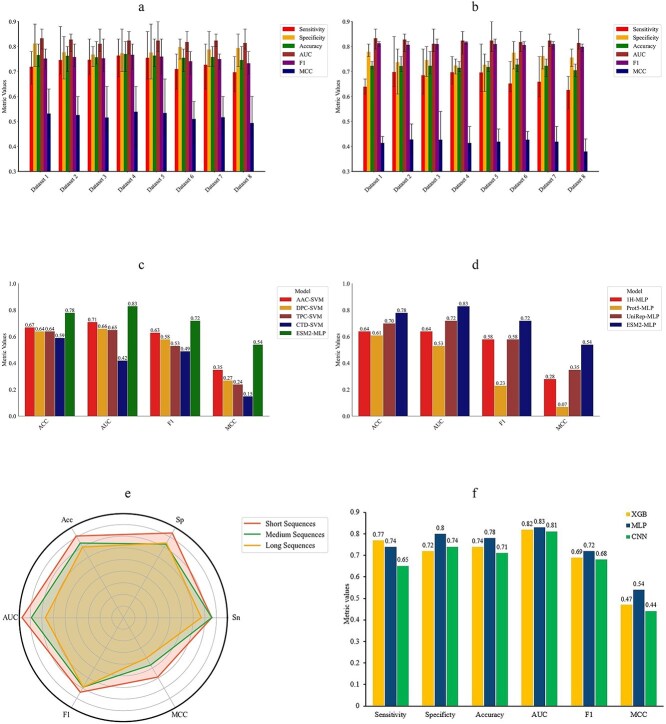
Overall evaluation of the model. Panels A and B show the average cross-validation and test SN, SP, ACC, AUC, F1, and MCC for models trained on Datasets 1 to 8, respectively. Error bars indicate the minimum and maximum values observed across cross-validation and test folds. Panels C and D compare the performance of the MLP-based ESM2_t36 model with models trained on traditional sequence-derived features, 1H encoding, ProtT5, and UniRep-derived embeddings. Panel E shows the comparison of the predictive performance of the MLP-based ESM2_t36 model across sequences of varying lengths. Panel F shows a performance comparison of the MLP classifier with other classifiers.

Across all eight training datasets, the MLP-based ESM2_t36 model demonstrated consistently high performance during cross-validation. The average sensitivity (SN) values ranged from 0.70 to 0.76, while specificity (SP) remained between 0.77 and 0.81. The accuracy (ACC) spanned 0.75–0.77, indicating robust predictive capability. The area under the ROC curve (AUC) consistently fell within the range of 0.81–0.83, underscoring the model’s high discriminative power. Additionally, F1 ranged from 0.73 to 0.77, and Matthews Correlation Coefficient (MCC) values ranged from 0.49 to 0.54, reflecting balanced performance across both positive and negative classes (Fig. 3a). The model’s performance on the test dataset was similarly stable, demonstrating strong generalization ability. Test SN ranged from 0.63 to 0.70, SP from 0.72 to 0.78, and ACC from 0.71 to 0.73. The AUC scores on test dataset remained high, typically between 0.80 and 0.82. Corresponding F1 ranged from 0.62 to 0.66, with MCC values averaging between 0.38 and 0.43 (Fig. 3b).

To enhance predictive robustness, we implemented an ensemble strategy by selecting the best-performing fold from each dataset and aggregating predictions using a majority vote. In cases of tie votes, the average predicted probability was used with a threshold of 0.5 to determine the final class label. The final ensemble model achieved an SN of 0.74, SP of 0.80, ACC of 0.78, AUC of 0.83, F1 of 0.72, and MCC of 0.54 on the test dataset (Table 2). Although the performance metrics of the ensemble model were comparable to the best individual model (Dataset 3, Table 2), the ensemble approach offers several methodological advantages. It enhances robustness and generalizability by aggregating predictions from multiple models and each trained on a distinct subset of the negative data. This strategy ensures that the entire negative dataset contributes to the learning process, thereby minimizing sampling bias and reducing model variance. Additionally, the ensemble’s majority voting mechanism augmented by probabilistic tie-breaking produces more stable and reliable predictions. Therefore, even with similar individual model performance, the ensemble method represents a more comprehensive and dependable solution, particularly in scenarios where multiple training datasets are employed to address class imbalance.

**Table 2:** Performance metrics of the best individual models and the ensemble model on the test dataset

**Dataset**	**SN**	**SP**	**ACC**	**AUC**	**F1**	**MCC**
1	0.63	0.81	0.74	0.81	0.65	0.44
2	0.72	0.78	0.76	0.82	0.69	0.49
3	0.74	0.80	0.78	0.82	0.72	0.54
4	0.73	0.75	0.74	0.82	0.68	0.47
5	0.69	0.77	0.74	0.83	0.67	0.46
6	0.64	0.82	0.75	0.82	0.66	0.46
7	0.72	0.77	0.75	0.82	0.68	0.48
8	0.68	0.76	0.73	0.80	0.66	0.43
Ensemble	0.74	0.80	0.78	0.82	0.72	0.54

#### Performance comparison of multilayer perceptron-based ESM2_t36 model with other ESM2 variants

We conducted a comprehensive evaluation of three ESM2 variants (t30, t33, and t36) to assess the trade-offs between computational efficiency and predictive performance using multiple metrics, including SN, SP, ACC, AUC, F1, and MCC.

Among the three variants, ESM2_t36 demonstrated the highest predictive performance, achieving an SN of 0.74, SP of 0.80, ACC of 0.78, AUC of 0.83, F1 of 0.72, and MCC of 0.54. However, this variant also required significantly more computational time for embedding generation, model training, and prediction. The ESM2_t33 variant yielded moderate predictive performance (SN: 0.72, SP: 0.75, ACC: 0.74, AUC: 0.80, F1: 0.67, MCC: 0.46) and exhibited moderate runtime requirements, offering a balanced trade-off between performance and efficiency. In contrast, the ESM2_t30 variant offered the fastest runtime but at the cost of reduced predictive performance (SN: 0.68, SP: 0.76, ACC: 0.73, AUC: 0.80, F1: 0.66, MCC: 0.43).

These results suggest that ESM2_t36 is the most suitable choice when predictive performance is the priority, whereas t30 and t33 may be more appropriate in scenarios where computational efficiency is critical and slight compromises in ACC are acceptable. The detailed comparison of the runtime and prediction performance of all three ESM2 variants is presented in Table 3. Considering the better predictive performance, we selected ESM2_t36 for further analysis.

**Table 3:** Runtime and predictive accuracy of different embedding generation strategies

**Embedding generation approaches**		**Run time**			**Metrics**					
		**embedding generation (sec/seq)**	**Model training (sec)**	**Making predictions (sec/seq)**	**SN**	**SP**	**ACC**	**AUC**	**F1**	**MCC**
iFeature	AAC	0.0017	40.59	0.0019	0.73	0.62	0.67	0.71	0.63	0.35
	DPC	0.0061	180.15	0.0062	0.65	0.64	0.64	0.66	0.58	0.27
	TPC	0.3851	28309.03	0.4021	0.55	0.69	0.64	0.65	0.53	0.24
	CTD	0.0179	118.30	0.0181	0.52	0.63	0.59	0.42	0.49	0.15
1H coding		0.0025	11403.35	0.0027	0.65	0.64	0.64	0.64	0.58	0.28
ESM2	t30	02.98	1265.71	3.10	0.68	0.76	0.73	0.80	0.66	0.43
	t33	06.03	1453.68	6.97	0.72	0.75	0.74	0.80	0.67	0.46
	t36	38.95	1323.03	40.02	0.74	0.80	0.78	083	0.72	0.54
Prot-t5		36.33	811.91	37.55	0.15	0.90	0.61	0.53	0.23	0.07
Unirep		2.97	1263.25	3.07	0.56	0.78	0.70	0.72	0.58	0.35

#### Performance comparison of multilayer perceptron-based ESM2_t36 model with other approaches

To evaluate the effectiveness of our MLP-based ESM2_t36 model, we conducted a comprehensive performance comparison with traditional sequence-based models, 1H encoding-based model, and other protein PLMs. The time for embedding generation, model training and prediction for each model was also counted (Table 3). The goal was to assess the added value of deep contextual representations learned by protein language models in identifying regulator proteins in LLPS.

First, we benchmarked the MLP-based ESM2_t36 model against traditional machine learning models utilizing sequence-derived features such as AAC, DPC, TPC, and CTD, coupled with an SVM classifier. The MLP-based ESM2_t36-based model consistently outperformed these traditional approaches across all evaluation metrics. Specifically, it achieved an ACC of 0.78, AUC of 0.83, F1 of 0.72, and MCC of 0.54. In contrast, the best-performing traditional model, based on AAC features, achieved an ACC of 0.67, AUC of 0.71, F1 of 0.63, and MCC of 0.35. These results highlight the superior capability of ESM2_t36 in capturing complex sequence-level patterns that are not easily encoded by handcrafted features (Fig. 3c).

We also compared the ESM2_t36-based model with a model trained using 1H encoding, a positional encoding technique. The 1H encoding-based model achieved an ACC of 0.64, AUC of 0.64, F1 of 0.58, and MCC of 0.28, which was notably lower than the ESM2_t36-based model’s prediction performance. This further supports the effectiveness of our approach in extracting biologically relevant information from protein sequences (Fig. 3d).

Finally, we evaluated the performance of our model against other state-of-the-art protein PLMs, including UniRep and ProtT5. The UniRep-based model achieved an ACC of 0.70, AUC of 0.72, F1 of 0.58, and MCC of 0.35, while the ProtT5-based model achieved an ACC of 0.61, AUC of 0.53, F1 of 0.23, and MCC of 0.07. In comparison, our MLP-based ESM2_t36 model clearly demonstrated superior performance across all metrics (Fig. 3d).

Overall, these results confirm that the deep contextual representations learned by ESM2_36 provide a significant advantage over both traditional sequence-based features, 1H encoding, and other PLM-based representations for the identification of regulator proteins in LLPS (Table 3). Considering this we consider MLP-based ESM2_t36 model as a final model for prediction of regulating LLPS.

#### Performance evaluation of multilayer perceptron-based ESM2_t36 model on sequence length

To assess the impact of sequence length on model performance, we categorized the test dataset into three groups: Group 1 (short sequences, ≤500 amino acids), Group 2 (medium sequences, 501–1000 amino acids), and Group 3 (long sequences, >1000 amino acids, up to 5008 residues). This grouping ensured sufficient sample sizes within each category, as the majority of sequences in the test dataset were under 2000 amino acids.

For short sequences, the model performs best, achieving a high SN of 0.76, SP of 0.84, ACC of 0.81, AUC of 0.87, F1 of 0.74, and MCC of 0.59, indicating strong overall performance. This group had the largest number of protein sequences, which may have contributed to the model’s better performance. The large dataset allowed the model to learn more effectively, potentially leading to better generalization. In contrast, for medium-length sequences, the performance slightly drops, with SN (0.76), SP (0.73), ACC (0.74), AUC (0.79), F1 (0.69), and MCC (0.47) all showing a decline except SN. The medium group contained fewer sequences than the short group, possibly limiting the model’s ability to generalize effectively on these sequences. The performance further diminishes for long sequences, where SN (0.67), SP (0.74), ACC (0.70), AUC (0.67), F1 (0.69), and MCC (0.40) all show a decline except SP and F1, indicating that longer sequences introduce more challenges for the model, likely due to increased complexity, fewer distinguishing features, or the relatively small number of protein sequences in this group (Fig. 3e).

This decline in performance can be attributed to several factors. Firstly, the short sequence group made up 70% of the test dataset, the medium-length group accounted for 18%, and the long sequence group only comprised 12%. This reduction in the number of protein sequences from the small to the large sequence group likely contributed to the observed performance decline. Additionally, the ESM2_t36 model may capture more local, global, and physicochemical information effectively from smaller sequences compared to larger sequences. Shorter sequences may have fewer complexities and clearer patterns, making them easier to classify, whereas longer sequences may introduce more noise or require more intricate feature extraction, which the model may struggle to learn effectively given the smaller dataset.

#### Performance comparison of the multilayer perceptron-based ESM2_t36 model with other classifiers

To identify the most effective model for identifying regulator proteins, we evaluated several classifiers, including XGB, MLP, and CNN. The performance metrics for these classifiers were as follows: XGB achieved an SN of 0.77, SP of 0.72, ACC of 0.74, AUC of 0.82, F1 of 0.69, and MCC of 0.47. Similarly, MLP achieved an SN of 0.74, SP of 0.80, ACC of 0.78, AUC of 0.83, F1 of 0.72, and MCC of 0.54, while CNN produced an SN of 0.65, SP of 0.74, ACC of 0.71, AUC of 0.81, F1 of 0.68, and MCC of 0.44. A comparison of these results is shown in Fig. 3f. Our analysis indicated that the MLP classifier achieved the highest SP, ACC, AUC, F1, and MCC, leading us to select the MLP-based model as the final approach for accurately identifying regulator proteins. Although the XGB classifier exhibited slightly higher SN, but its SP, ACC, AUC, F1, and MCC were relatively low. Consequently, we selected the MLP-based ESM2_t36 model as our final model. Detailed results are presented in the supplementary file.

### Model interpretation and analysis

#### SHAP feature analysis

For functional interpretation, amino acids were first classified into four groups based on their physicochemical properties: (i) hydrophobic residues (A, V, I, L, M, F, W, P), which promote compact and stable folded structures, are predominantly enriched in non-regulator proteins; (ii) hydrophilic residues (S, T, N, Q, Y), which mediate dynamic, reversible interactions and are strongly associated with LLPS regulators; (iii) charged residues (R, K, D, E), which facilitate electrostatic interactions and exhibit a positive correlation with LLPS regulation; and (iv) other residues (C, G, H), which show moderate enrichment in LLPS proteins and contribute to the modulation and context-dependent stability of biomolecular condensates.

**Figure 4:**
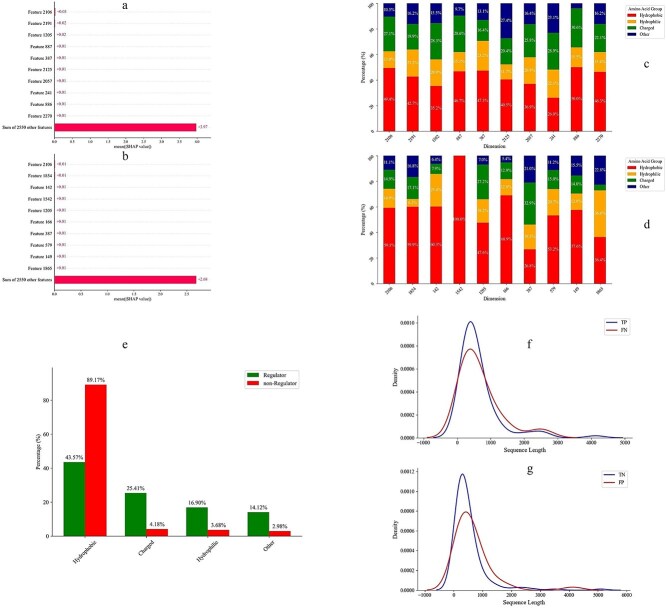
Interpretation and analysis of the model. Panels A and B present the SHAP analysis, highlighting the top ten feature dimensions that contribute most to the model’s predictions for the positive and negative classes, respectively. Panels C and D illustrate the distribution of amino acid residues across the top ten most important feature dimensions contributing to the model’s decision for the positive and negative classes. Panel E shows the average distribution of amino acid residues across the top 10 most important feature dimensions contributing to the model’s decisions for both positive and negative classes. Panels F and G illustrate the comparison of sequence length distributions between TP and FN protein sequences, and between TN and FP protein sequences.

To elucidate the molecular features that regulate LLPS, we employed SHAP (SHapley Additive exPlanations) [71, 72] analysis to identify biologically relevant features that drive model predictions. For computational efficiency, we randomly selected a subset comprising 30 regulator proteins and 30 non-regulator proteins from the test dataset. SHAP analysis was then applied to extract the top 10 most discriminative feature dimensions for each class, which were mapped to the respective protein sequences to assess residue-level contributions. The top 10 SHAP-derived feature dimensions for LLPS regulator proteins were identified as 2106, 2191, 1205, 887, 387, 1225, 2027, 241, 886, and 2270. These dimensions were primarily enriched in residues associated with LLPS regulators. Similarly, the top 10 SHAP-derived feature dimensions for non-regulator proteins were identified as 2106, 1854, 142, 1542, 1205, 166, 387, 579, 149, and 1865 revealing a contrasting distribution with a higher prevalence of residues characteristic of non-LLPS regulators (Fig. 4a–d). Additionally, the SHAP analysis identified three common dimensions (2106, 1205, and 387) that were important for both LLPS regulators and non-regulators.

To enhance the interpretability of our results, we computed the average contribution of each amino acid group across the top 10 most influential feature dimensions, as identified by SHAP analysis, for both regulator and non-regulator proteins. A clear compositional divergence was observed between the two classes. In non-regulator proteins, hydrophobic residues were predominant, accounting for ~89.17% of the contributing features, followed by charged (4.18%), hydrophilic (3.68%), and other residues (2.98%). In contrast, LLPS regulator proteins displayed a more balanced distribution: 43.57% hydrophobic, 25.41% charged, 16.90% hydrophilic, and 14.12% other residues (Fig. 4e).

These results suggest that hydrophobic residues play a key role in distinguishing non-regulators in LLPS, likely due to their contribution to structural stability. In contrast, LLPS regulators rely more heavily on charged and hydrophilic residues, which are typically associated with intrinsic disorder and multivalent interactions which are key features driving phase separation.

#### Analysis of misclassified sequences

To investigate potential reasons for the model’s misclassification of false negatives (FN) and false positives (FP), we conducted an analysis focusing on biologically relevant properties associated with LLPS regulator and non-regulator proteins. Specifically, we examined sequence length, disordered content, low-complexity content, hydrophobicity, and charge distribution (Table 4). Our experimental design involved comparing each feature between true positives (TP) and FN, and between true negatives (TN) and FP, aiming to determine whether significant differences in these properties might have contributed to the observed misclassifications. Given the imbalance between correctly and incorrectly classified proteins, we calculated and compared the average values for all properties.

**Table 4:** Distribution of key physicochemical features in misclassified protein sequences

**Physicochemical properties**	**TP**	**FN**	**TN**	**FP**
Hydrophobicity	−0.51	−0.49	−0.34	−0.41
Disordered content	0.75	0.75	0.74	0.75
Low complexity content	0.53	0.52	0.52	0.52
Positively charge residues	0.16	0.15	0.15	0.14
Negatively charge residues	0.13	0.13	0.12	0.13

Our analysis revealed that the average sequence lengths of both FN and FP proteins were shorter than those of TP and TN, respectively (Fig. 4f and g). For hydrophobicity, we employed the Kyte–Doolittle scale, which assigns each amino acid a hydropathy index based on its tendency to be hydrophobic or hydrophilic [73]. TP proteins displayed lower average hydrophobicity compared to FN, and similarly, FP proteins were less hydrophobic than TN. This suggests that increased hydrophobicity may have contributed to the misclassification of FN as non-regulators and lower hydrophobicity may have contributed to the misclassification of FP as regulators. Disordered content was assessed based on the frequency of amino acids commonly associated with intrinsic disorder regions (P, G, S, Q, E, A, R, K, N, T, D, L). FN and TP sequences exhibited comparable disorder content, whereas FP proteins showed slightly elevated disorder content relative to TN, potentially leading the model to incorrectly classify them as regulators. Low-complexity content was calculated using amino acids typically enriched in LLPS-related low-complexity regions (A, G, S, I, P, Y, Q, N, F, R). FN proteins showed slightly lower low-complexity content than TP, which may have contributed to their misclassification, as LLPS-regulating proteins are generally associated with increased low-complexity regions. In contrast, FP and TN sequences had similar low-complexity content. Charge distribution was analyzed by computing the fraction of positively charged residues (R, K, H) and negatively charged residues (D, E). FN proteins had marginally lower levels of positively charged residues compared to TP, potentially influencing their misclassification. However, FP sequences also had reduced positive charge relative to TN, which does not align with the hypothesis that regulator proteins have higher charge content which suggested that the model may rely on additional features beyond charge distribution. Regarding negative charges, FN and TP exhibited similar levels, whereas FP proteins had a higher negative charge content than TN, possibly leading to their incorrect classification as regulators.

Based on this feature-level analysis, the misclassification of FN and FP can be attributed to subtle but biologically meaningful deviations in key protein properties compared to correctly classified samples. FN proteins tended to exhibit higher hydrophobicity, shorter sequence lengths, slightly lower low-complexity content, and reduced positive charge relative to TP proteins, which may have caused the model to overlook their regulator potential in LLPS. These traits differ from those typically enriched in known regulators, suggesting the model may have learned to associate specific biophysical signatures such as, lower hydrophobicity, higher low-complexity and dis-ordered content, and greater positive charge with positive classification.

Interestingly, our sequence length-based analysis revealed an apparent contradiction: while prior results indicated that the model performs well on shorter sequences overall, many misclassified proteins both FN and FP were also characterized by shorter lengths. This inconsistency suggests that sequence length alone is not a sufficient determinant of classification performance. Instead, it implies that the interplay between sequence length and other features such as low-complexity regions, disorder content, or charge distribution, which may influence the model’s ability to correctly classify proteins. In other words, some shorter sequences may lack other defining characteristics of LLPS regulators or non-regulators may be inherently more challenging for the model to classify accurately. Conversely, FP proteins shared certain characteristics with regulators, such as elevated disordered content, negative charge, and lower hydrophobicity, which potentially misleading the model. Although their low-complexity and charge distributions were similar to TN proteins, their increased disorder and higher negative charge may have skewed the model’s decision boundary, resulting in misclassification as regulators. Overall, these findings indicate that the model’s predictions may be sensitive to nuanced variations in hydrophobicity, disorder content, and charge, and that refining feature representation or integrating additional contextual features may enhance classification accuracy.

## Conclusion

In this study, we developed an artificial intelligence-based framework for the identification of regulator proteins involved in LLPS. A high-quality dataset comprising 913 experimentally validated regulators and 6584 non-regulators was curated, and protein embeddings were extracted using the ESM2_t36 PLM. An MLP classifier was then trained using balanced ensemble learning and achieved robust performance across multiple metrics (ACC: 0.78; AUC: 83; F1: 72; MCC: 0.54). To evaluate the effectiveness of our method, we conducted comparative analyses against traditional sequence-based features, 1H encoding, and other protein language models. Our ESM2_t36-based approach consistently outperformed these baselines, underscoring the advantages of leveraging deep contextual representations for this task. Furthermore, model interpretability was enhanced through SHAP, which revealed that predicted LLPS regulators are enriched in charged and hydrophilic residues, which are known to facilitate phase separation. Additionally, misclassification analysis indicated that factors such as sequence length and specific biophysical properties may influence prediction accuracy, offering further insights into the model’s behavior and the complexity of LLPS regulation. Overall, this work represents one of the first systematic attempts to predict LLPS regulator proteins using deep learning and contextual sequence embeddings. The proposed framework not only advances computational LLPS research but also provides a scalable tool for applications in synthetic biology, drug discovery, and disease-related studies.

**Key Points**

- A high-quality dataset was curated to train the model for the identification of regulator proteins in liquid–liquid phase separation (LLPS).
- The pretrained protein language model, ESM2, was utilized to extract comprehensive features, including protein sequences, structural information, and evolutionary patterns.
- Class imbalance was addressed by partitioning the negative dataset into smaller subsets, ensuring a balanced representation during model training.
- An ensemble modeling strategy was employed to integrate the contributions of all trained models, enhancing the robustness and accuracy of the final predictions.
- To facilitate accessibility, the source code of the model has been made publicly available.

## Supplementary Material

supplimentary_file_bbaf313

## Data Availability

All source code and datasets are publicly available at: https://github.com/bioplusAI/LLPS_regulators_pred.
