# Supplementary material for: An artificial intelligence-based approach for identifying the proteins regulating liquid–liquid phase separation
Source: Brief Bioinform. 2025 Jul 9;26(4):bbaf313. doi: 10.1093/bib/bbaf313 (PMC12239617; doi:10.1093/bib/bbaf313)
Supplement: supplimentary_file_bbaf313 [file supplimentary_file_bbaf313.docx]

**An artificial intelligence-based approach for identifying the proteins regulating liquid-liquid phase separation**

**Supplementary file**

**Results using MLP**

**Table: S1** Sensitivity, specificity, and accuracy on dataset 1

| **Folds** | **Cross-validation** | | | | | | **Test dataset** | | | | | |
| --- | --- | --- | --- | --- | --- | --- | --- | --- | --- | --- | --- | --- |
|  | **SN** | **SP** | **ACC** | **AUC** | **F1** | **MCC** | **SN** | **SP** | **ACC** | **AUC** | **F1** | **MCC** |
| 1 | 0.67 | 0.89 | 0.77 | 0.85 | 0.76 | 0.56 | 0.67 | 0.77 | 0.73 | 0.81 | 0.66 | 0.43 |
| 2 | 0.75 | 0.72 | 0.74 | 0.79 | 0.75 | 0.47 | 0.66 | 0.76 | 0.72 | 0.81 | 0.64 | 0.41 |
| 3 | 0.65 | 0.85 | 0.76 | 0.83 | 0.71 | 0.51 | 0.61 | 0.78 | 0.71 | 0.82 | 0.62 | 0.39 |
| 4 | 0.68 | 0.80 | 0.74 | 0.81 | 0.72 | 0.48 | 0.62 | 0.79 | 0.72 | 0.82 | 0.63 | 0.41 |
| 5 | 0.74 | 0.87 | 0.81 | 0.87 | 0.79 | 0.61 | 0.65 | 0.79 | 0.73 | 0.81 | 0.65 | 0.43 |
| 6 | 0.73 | 0.8 | 0.76 | 0.82 | 0.76 | 0.53 | 0.65 | 0.78 | 0.73 | 0.81 | 0.65 | 0.43 |
| 7 | 0.74 | 0.88 | 0.81 | 0.86 | 0.79 | 0.63 | 0.65 | 0.76 | 0.72 | 0.8 | 0.64 | 0.41 |
| 8 | 0.75 | 0.76 | 0.76 | 0.84 | 0.76 | 0.51 | 0.63 | 0.77 | 0.71 | 0.82 | 0.63 | 0.39 |
| 9 | 0.70 | 0.74 | 0.72 | 0.80 | 0.71 | 0.44 | 0.62 | 0.78 | 0.72 | 0.82 | 0.63 | 0.4 |
| 10 | 0.78 | 0.81 | 0.79 | 0.86 | 0.77 | 0.58 | 0.63 | 0.81 | 0.74 | 0.81 | 0.65 | 0.44 |
| **Mean** | 0.72 | 0.81 | 0.77 | 0.83 | 0.75 | 0.53 | 0.64 | 0.78 | 0.72 | 0.81 | 0.64 | 0.41 |

**Table: S2** Sensitivity, specificity, and accuracy on dataset 2

| **Folds** | **Cross-validation** | | | | | | **Test dataset** | | | | | |
| --- | --- | --- | --- | --- | --- | --- | --- | --- | --- | --- | --- | --- |
|  | **SN** | **SP** | **ACC** | **AUC** | **F1** | **MCC** | **SN** | **SP** | **ACC** | **AUC** | **F1** | **MCC** |
| 1 | 0.71 | 0.84 | 0.77 | 0.84 | 0.77 | 0.55 | 0.72 | 0.78 | 0.76 | 0.82 | 0.69 | 0.49 |
| 2 | 0.73 | 0.76 | 0.74 | 0.83 | 0.75 | 0.49 | 0.67 | 0.79 | 0.74 | 0.81 | 0.67 | 0.46 |
| 3 | 0.69 | 0.8 | 0.75 | 0.82 | 0.72 | 0.50 | 0.68 | 0.75 | 0.72 | 0.79 | 0.65 | 0.43 |
| 4 | 0.74 | 0.78 | 0.76 | 0.81 | 0.75 | 0.51 | 0.66 | 0.72 | 0.70 | 0.81 | 0.62 | 0.37 |
| 5 | 0.77 | 0.83 | 0.80 | 0.85 | 0.79 | 0.60 | 0.64 | 0.75 | 0.71 | 0.80 | 0.62 | 0.38 |
| 6 | 0.73 | 0.67 | 0.70 | 0.78 | 0.72 | 0.40 | 0.77 | 0.72 | 0.74 | 0.81 | 0.69 | 0.48 |
| 7 | 0.77 | 0.79 | 0.78 | 0.81 | 0.77 | 0.56 | 0.65 | 0.73 | 0.70 | 0.81 | 0.62 | 0.37 |
| 8 | 0.88 | 0.67 | 0.78 | 0.85 | 0.81 | 0.57 | 0.84 | 0.61 | 0.70 | 0.79 | 0.68 | 0.44 |
| 9 | 0.72 | 0.81 | 0.77 | 0.84 | 0.76 | 0.54 | 0.67 | 0.74 | 0.71 | 0.82 | 0.64 | 0.41 |
| 10 | 0.72 | 0.82 | 0.78 | 0.85 | 0.74 | 0.54 | 0.68 | 0.78 | 0.74 | 0.81 | 0.67 | 0.45 |
| **Mean** | 0.75 | 0.78 | 0.76 | 0.83 | 0.76 | 0.53 | 0.70 | 0.74 | 0.72 | 0.81 | 0.66 | 0.43 |

**Table: S3** Sensitivity, specificity, and accuracy on dataset 3

| **Folds** | **Cross-validation** | | | | | | **Test dataset** | | | | | |
| --- | --- | --- | --- | --- | --- | --- | --- | --- | --- | --- | --- | --- |
|  | **SN** | **SP** | **ACC** | **AUC** | **F1** | **MCC** | **SN** | **SP** | **ACC** | **AUC** | **F1** | **MCC** |
| 1 | 0.71 | 0.78 | 0.74 | 0.79 | 0.75 | 0.49 | 0.72 | 0.77 | 0.75 | 0.82 | 0.69 | 0.48 |
| 2 | 0.77 | 0.75 | 0.76 | 0.83 | 0.77 | 0.52 | 0.61 | 0.74 | 0.69 | 0.8 | 0.60 | 0.35 |
| 3 | 0.69 | 0.77 | 0.73 | 0.79 | 0.7 | 0.46 | 0.64 | 0.74 | 0.70 | 0.8 | 0.62 | 0.37 |
| 4 | 0.69 | 0.80 | 0.74 | 0.80 | 0.73 | 0.49 | 0.64 | 0.78 | 0.73 | 0.83 | 0.64 | 0.42 |
| 5 | 0.74 | 0.76 | 0.75 | 0.80 | 0.74 | 0.50 | 0.65 | 0.74 | 0.71 | 0.81 | 0.63 | 0.39 |
| 6 | 0.83 | 0.80 | 0.82 | 0.87 | 0.83 | 0.64 | 0.79 | 0.68 | 0.72 | 0.81 | 0.68 | 0.46 |
| 7 | 0.81 | 0.80 | 0.81 | 0.84 | 0.8 | 0.61 | 0.61 | 0.77 | 0.71 | 0.81 | 0.61 | 0.38 |
| 8 | 0.74 | 0.75 | 0.74 | 0.78 | 0.75 | 0.49 | 0.68 | 0.76 | 0.73 | 0.79 | 0.66 | 0.44 |
| 9 | 0.74 | 0.72 | 0.73 | 0.81 | 0.73 | 0.46 | 0.77 | 0.68 | 0.71 | 0.81 | 0.67 | 0.44 |
| 10 | 0.75 | 0.75 | 0.75 | 0.8 | 0.73 | 0.50 | 0.74 | 0.8 | 0.78 | 0.82 | 0.72 | 0.54 |
| **Mean** | 0.75 | 0.77 | 0.76 | 0.81 | 0.75 | 0.52 | 0.69 | 0.75 | 0.72 | 0.81 | 0.65 | 0.43 |

**Table: S4** Sensitivity, specificity, and accuracy on dataset 4

| **Folds** | **Cross-validation** | | | | | | **Test dataset** | | | | | |
| --- | --- | --- | --- | --- | --- | --- | --- | --- | --- | --- | --- | --- |
|  | **SN** | **SP** | **ACC** | **AUC** | **F1** | **MCC** | **SN** | **SP** | **ACC** | **AUC** | **F1** | **MCC** |
| 1 | 0.68 | 0.73 | 0.7 | 0.77 | 0.71 | 0.4 | 0.66 | 0.73 | 0.71 | 0.82 | 0.63 | 0.39 |
| 2 | 0.79 | 0.75 | 0.77 | 0.83 | 0.78 | 0.54 | 0.65 | 0.73 | 0.7 | 0.82 | 0.63 | 0.38 |
| 3 | 0.77 | 0.80 | 0.79 | 0.85 | 0.77 | 0.57 | 0.76 | 0.73 | 0.74 | 0.82 | 0.69 | 0.48 |
| 4 | 0.72 | 0.75 | 0.74 | 0.81 | 0.73 | 0.48 | 0.69 | 0.71 | 0.7 | 0.82 | 0.64 | 0.39 |
| 5 | 0.82 | 0.82 | 0.82 | 0.86 | 0.81 | 0.64 | 0.73 | 0.71 | 0.72 | 0.82 | 0.66 | 0.43 |
| 6 | 0.71 | 0.87 | 0.79 | 0.85 | 0.78 | 0.59 | 0.73 | 0.75 | 0.74 | 0.82 | 0.68 | 0.47 |
| 7 | 0.78 | 0.79 | 0.79 | 0.84 | 0.78 | 0.57 | 0.66 | 0.73 | 0.71 | 0.81 | 0.63 | 0.39 |
| 8 | 0.83 | 0.7 | 0.77 | 0.82 | 0.79 | 0.54 | 0.71 | 0.69 | 0.70 | 0.81 | 0.64 | 0.39 |
| 9 | 0.78 | 0.72 | 0.75 | 0.79 | 0.76 | 0.5 | 0.73 | 0.72 | 0.72 | 0.82 | 0.66 | 0.43 |
| 10 | 0.76 | 0.8 | 0.78 | 0.82 | 0.76 | 0.56 | 0.65 | 0.74 | 0.71 | 0.81 | 0.63 | 0.39 |
| **Mean** | 0.76 | 0.77 | 0.77 | 0.82 | 0.77 | 0.54 | 0.70 | 0.72 | 0.72 | 0.82 | 0.65 | 0.41 |

**Table: S5** Sensitivity, specificity, and accuracy on dataset 5

| **Folds** | **Cross-validation** | | | | | | **Test dataset** | | | | | |
| --- | --- | --- | --- | --- | --- | --- | --- | --- | --- | --- | --- | --- |
|  | **SN** | **SP** | **ACC** | **AUC** | **F1** | **MCC** | **SN** | **SP** | **ACC** | **AUC** | **F1** | **MCC** |
| 1 | 0.67 | 0.81 | 0.73 | 0.80 | 0.73 | 0.48 | 0.65 | 0.76 | 0.72 | 0.81 | 0.64 | 0.41 |
| 2 | 0.81 | 0.78 | 0.79 | 0.86 | 0.80 | 0.59 | 0.80 | 0.68 | 0.73 | 0.81 | 0.69 | 0.47 |
| 3 | 0.68 | 0.77 | 0.72 | 0.78 | 0.69 | 0.45 | 0.62 | 0.74 | 0.70 | 0.81 | 0.61 | 0.36 |
| 4 | 0.81 | 0.70 | 0.76 | 0.82 | 0.77 | 0.52 | 0.74 | 0.67 | 0.70 | 0.80 | 0.65 | 0.40 |
| 5 | 0.86 | 0.67 | 0.76 | 0.80 | 0.78 | 0.54 | 0.81 | 0.62 | 0.70 | 0.79 | 0.67 | 0.43 |
| 6 | 0.75 | 0.75 | 0.75 | 0.79 | 0.76 | 0.50 | 0.69 | 0.77 | 0.74 | 0.83 | 0.67 | 0.45 |
| 7 | 0.77 | 0.80 | 0.79 | 0.85 | 0.78 | 0.57 | 0.65 | 0.75 | 0.71 | 0.82 | 0.64 | 0.40 |
| 8 | 0.77 | 0.89 | 0.83 | 0.9 | 0.83 | 0.67 | 0.69 | 0.75 | 0.73 | 0.80 | 0.66 | 0.43 |
| 9 | 0.68 | 0.81 | 0.74 | 0.82 | 0.72 | 0.49 | 0.62 | 0.76 | 0.71 | 0.80 | 0.62 | 0.38 |
| 10 | 0.75 | 0.78 | 0.77 | 0.82 | 0.74 | 0.53 | 0.69 | 0.77 | 0.74 | 0.83 | 0.67 | 0.46 |
| **Mean** | 0.76 | 0.78 | 0.76 | 0.82 | 0.76 | 0.53 | 0.70 | 0.73 | 0.72 | 0.81 | 0.65 | 0.42 |

**Table: S6** Sensitivity, specificity, and accuracy on dataset 6

| **Folds** | **Cross-validation** | | | | | | **Test dataset** | | | | | |
| --- | --- | --- | --- | --- | --- | --- | --- | --- | --- | --- | --- | --- |
|  | **SN** | **SP** | **ACC** | **AUC** | **F1** | **MCC** | **SN** | **SP** | **ACC** | **AUC** | **F1** | **MCC** |
| 1 | 0.63 | 0.78 | 0.70 | 0.78 | 0.70 | 0.41 | 0.65 | 0.8 | 0.75 | 0.82 | 0.66 | 0.46 |
| 2 | 0.71 | 0.83 | 0.77 | 0.86 | 0.76 | 0.54 | 0.65 | 0.8 | 0.74 | 0.81 | 0.65 | 0.45 |
| 3 | 0.64 | 0.83 | 0.74 | 0.81 | 0.69 | 0.47 | 0.65 | 0.77 | 0.72 | 0.8 | 0.64 | 0.42 |
| 4 | 0.70 | 0.76 | 0.73 | 0.80 | 0.72 | 0.46 | 0.64 | 0.82 | 0.75 | 0.82 | 0.66 | 0.46 |
| 5 | 0.71 | 0.83 | 0.78 | 0.82 | 0.75 | 0.55 | 0.63 | 0.76 | 0.71 | 0.81 | 0.62 | 0.39 |
| 6 | 0.67 | 0.82 | 0.74 | 0.8 | 0.73 | 0.49 | 0.65 | 0.78 | 0.73 | 0.80 | 0.64 | 0.42 |
| 7 | 0.77 | 0.79 | 0.78 | 0.82 | 0.77 | 0.56 | 0.62 | 0.79 | 0.73 | 0.80 | 0.63 | 0.42 |
| 8 | 0.77 | 0.75 | 0.76 | 0.83 | 0.77 | 0.52 | 0.66 | 0.77 | 0.73 | 0.81 | 0.65 | 0.43 |
| 9 | 0.76 | 0.81 | 0.79 | 0.84 | 0.78 | 0.58 | 0.74 | 0.71 | 0.72 | 0.79 | 0.67 | 0.44 |
| 10 | 0.74 | 0.78 | 0.76 | 0.82 | 0.74 | 0.52 | 0.63 | 0.75 | 0.70 | 0.80 | 0.62 | 0.38 |
| **Mean** | 0.71 | 0.80 | 0.76 | 0.82 | 0.74 | 0.51 | 0.65 | 0.78 | 0.73 | 0.81 | 0.64 | 0.43 |

**Table: S7** Sensitivity, specificity, and accuracy on dataset 7

| **Folds** | **Cross-validation** | | | | | | **Test dataset** | | | | | |
| --- | --- | --- | --- | --- | --- | --- | --- | --- | --- | --- | --- | --- |
|  | **SN** | **SP** | **ACC** | **AUC** | **F1** | **MCC** | **SN** | **SP** | **ACC** | **AUC** | **F1** | **MCC** |
| 1 | 0.63 | 0.82 | 0.72 | 0.8 | 0.71 | 0.46 | 0.67 | 0.76 | 0.73 | 0.81 | 0.65 | 0.43 |
| 2 | 0.71 | 0.83 | 0.77 | 0.85 | 0.76 | 0.54 | 0.60 | 0.79 | 0.72 | 0.81 | 0.62 | 0.39 |
| 3 | 0.73 | 0.86 | 0.80 | 0.85 | 0.77 | 0.60 | 0.61 | 0.72 | 0.68 | 0.79 | 0.59 | 0.33 |
| 4 | 0.71 | 0.74 | 0.72 | 0.81 | 0.72 | 0.45 | 0.65 | 0.78 | 0.73 | 0.82 | 0.65 | 0.43 |
| 5 | 0.71 | 0.81 | 0.76 | 0.83 | 0.74 | 0.52 | 0.72 | 0.77 | 0.75 | 0.82 | 0.68 | 0.48 |
| 6 | 0.71 | 0.75 | 0.73 | 0.80 | 0.74 | 0.46 | 0.66 | 0.80 | 0.75 | 0.81 | 0.67 | 0.47 |
| 7 | 0.81 | 0.72 | 0.76 | 0.82 | 0.77 | 0.53 | 0.76 | 0.71 | 0.73 | 0.81 | 0.68 | 0.45 |
| 8 | 0.77 | 0.74 | 0.76 | 0.83 | 0.77 | 0.51 | 0.66 | 0.76 | 0.72 | 0.81 | 0.65 | 0.42 |
| 9 | 0.71 | 0.84 | 0.78 | 0.82 | 0.76 | 0.55 | 0.61 | 0.76 | 0.70 | 0.81 | 0.61 | 0.37 |
| 10 | 0.78 | 0.77 | 0.78 | 0.83 | 0.76 | 0.55 | 0.65 | 0.77 | 0.72 | 0.81 | 0.64 | 0.42 |
| **Mean** | 0.73 | 0.79 | 0.76 | 0.82 | 0.75 | 0.52 | 0.66 | 0.76 | 0.72 | 0.81 | 0.64 | 0.42 |

**Table: S8** Sensitivity, specificity, and accuracy on dataset 8

| **Folds** | **Cross-validation** | | | | | | **Test dataset** | | | | | |
| --- | --- | --- | --- | --- | --- | --- | --- | --- | --- | --- | --- | --- |
|  | **SN** | **SP** | **ACC** | **AUC** | **F1** | **MCC** | **SN** | **SP** | **ACC** | **AUC** | **F1** | **MCC** |
| 1 | 0.64 | 0.79 | 0.71 | 0.79 | 0.71 | 0.44 | 0.68 | 0.74 | 0.72 | 0.81 | 0.65 | 0.42 |
| 2 | 0.70 | 0.83 | 0.76 | 0.81 | 0.76 | 0.53 | 0.58 | 0.79 | 0.71 | 0.81 | 0.61 | 0.38 |
| 3 | 0.62 | 0.84 | 0.74 | 0.79 | 0.69 | 0.47 | 0.64 | 0.77 | 0.72 | 0.80 | 0.63 | 0.40 |
| 4 | 0.74 | 0.72 | 0.73 | 0.79 | 0.73 | 0.46 | 0.63 | 0.72 | 0.68 | 0.80 | 0.60 | 0.34 |
| 5 | 0.73 | 0.75 | 0.74 | 0.81 | 0.73 | 0.47 | 0.65 | 0.77 | 0.72 | 0.80 | 0.64 | 0.41 |
| 6 | 0.62 | 0.83 | 0.72 | 0.82 | 0.70 | 0.46 | 0.6 | 0.77 | 0.70 | 0.80 | 0.61 | 0.37 |
| 7 | 0.74 | 0.85 | 0.8 | 0.87 | 0.78 | 0.6 | 0.68 | 0.76 | 0.73 | 0.80 | 0.66 | 0.43 |
| 8 | 0.76 | 0.78 | 0.77 | 0.86 | 0.78 | 0.54 | 0.6 | 0.73 | 0.68 | 0.79 | 0.59 | 0.33 |
| 9 | 0.71 | 0.75 | 0.73 | 0.81 | 0.73 | 0.46 | 0.65 | 0.73 | 0.70 | 0.80 | 0.63 | 0.38 |
| 10 | 0.71 | 0.8 | 0.76 | 0.79 | 0.72 | 0.51 | 0.55 | 0.78 | 0.69 | 0.78 | 0.58 | 0.34 |
| **Mean** | 0.70 | 0.79 | 0.75 | 0.81 | 0.73 | 0.49 | 0.63 | 0.76 | 0.71 | 0.80 | 0.62 | 0.38 |

**Table S9:** 10-fold Cross-validation and test sensitivity, specificity, accuracy, AUC, F1-score, and MCC for datasets 1 to 8 using ESM2_t33.

| **Datasets** | **Cross-validation** | | | | | | **Test dataset** | | | | | |
| --- | --- | --- | --- | --- | --- | --- | --- | --- | --- | --- | --- | --- |
|  | **SN** | **SP** | **ACC** | **AUC** | **F1** | **MCC** | **SN** | **SP** | **ACC** | **AUC** | **F1** | **MCC** |
| 1 | 0.74 | 0.77 | 0.75 | 0.82 | 0.75 | 0.51 | 0.73 | 0.73 | 0.73 | 0.81 | 0.67 | 0.44 |
| 2 | 0.71 | 0.8 | 0.75 | 0.81 | 0.74 | 0.51 | 0.69 | 0.74 | 0.72 | 0.8 | 0.65 | 0.42 |
| 3 | 0.73 | 0.75 | 0.74 | 0.8 | 0.74 | 0.48 | 0.67 | 0.72 | 0.7 | 0.79 | 0.63 | 0.39 |
| 4 | 0.74 | 0.78 | 0.76 | 0.82 | 0.75 | 0.52 | 0.69 | 0.73 | 0.71 | 0.81 | 0.64 | 0.41 |
| 5 | 0.74 | 0.77 | 0.76 | 0.81 | 0.75 | 0.51 | 0.69 | 0.72 | 0.71 | 0.8 | 0.64 | 0.4 |
| 6 | 0.72 | 0.78 | 0.75 | 0.81 | 0.74 | 0.5 | 0.67 | 0.74 | 0.71 | 0.79 | 0.64 | 0.4 |
| 7 | 0.72 | 0.77 | 0.75 | 0.81 | 0.74 | 0.5 | 0.68 | 0.72 | 0.71 | 0.79 | 0.64 | 0.4 |
| 8 | 0.76 | 0.76 | 0.76 | 0.81 | 0.76 | 0.52 | 0.72 | 0.7 | 0.71 | 0.79 | 0.65 | 0.41 |
| **Ensemble** | 0.72 | 0.75 | 0.74 | 0.80 | 0.67 | 0.46 |  |  |  |  |  |  |

**Table S10:** 10-fold Cross-validation and test sensitivity, specificity, accuracy, AUC, F1-score, and MCC for datasets 1 to 8 using ESM_t30.

| **Datasets** | **Cross-validation** | | | | | | **Test dataset** | | | | | |
| --- | --- | --- | --- | --- | --- | --- | --- | --- | --- | --- | --- | --- |
|  | **SN** | **SP** | **ACC** | **AUC** | **F1** | **MCC** | **SN** | **SP** | **ACC** | **AUC** | **F1** | **MCC** |
| 1 | 0.78 | 0.74 | 0.81 | 0.73 | 0.48 | 0.7 | 0.7 | 0.7 | 0.79 | 0.64 | 0.39 | 0.7 |
| 2 | 0.73 | 0.76 | 0.75 | 0.81 | 0.74 | 0.5 | 0.72 | 0.67 | 0.69 | 0.79 | 0.64 | 0.38 |
| 3 | 0.73 | 0.72 | 0.73 | 0.78 | 0.72 | 0.45 | 0.71 | 0.68 | 0.69 | 0.79 | 0.64 | 0.38 |
| 4 | 0.72 | 0.76 | 0.74 | 0.79 | 0.73 | 0.48 | 0.69 | 0.71 | 0.7 | 0.79 | 0.64 | 0.4 |
| 5 | 0.75 | 0.74 | 0.74 | 0.8 | 0.74 | 0.49 | 0.71 | 0.69 | 0.69 | 0.78 | 0.64 | 0.38 |
| 6 | 0.7 | 0.77 | 0.73 | 0.79 | 0.72 | 0.47 | 0.67 | 0.71 | 0.7 | 0.78 | 0.63 | 0.38 |
| 7 | 0.71 | 0.79 | 0.74 | 0.79 | 0.73 | 0.49 | 0.7 | 0.69 | 0.7 | 0.77 | 0.63 | 0.38 |
| 8 | 0.75 | 0.73 | 0.74 | 0.78 | 0.74 | 0.48 | 0.69 | 0.68 | 0.68 | 0.76 | 0.62 | 0.36 |
| **Ensemble** | 0.68 | 0.76 | 0.73 | 0.80 | 0.66 | 0.43 |  |  |  |  |  |  |

**Table S11:** 10-fold Cross-validation and test sensitivity, specificity, accuracy, AUC, F1-score, and MCC for datasets 1 to 8 using XGB.

| **Datasets** | **Cross-validation** | | | | | | **Test dataset** | | | | | |
| --- | --- | --- | --- | --- | --- | --- | --- | --- | --- | --- | --- | --- |
|  | **SN** | **SP** | **ACC** | **AUC** | **F1** | **MCC** | **SN** | **SP** | **ACC** | **AUC** | **F1** | **MCC** |
| 1 | 0.71 | 0.69 | 0.70 | 0.78 | 0.70 | 0.41 | 0.81 | 0.67 | 0.70 | 0.77 | 0.65 | 0.41 |
| 2 | 0.73 | 0.73 | 0.73 | 0.78 | 0.73 | 0.46 | 0.81 | 0.67 | 0.70 | 0.77 | 0.64 | 0.40 |
| 3 | 0.71 | 0.70 | 0.70 | 0.76 | 0.71 | 0.41 | 0.81 | 0.67 | 0.70 | 0.77 | 0.65 | 0.39 |
| 4 | 0.71 | 0.71 | 0.71 | 0.78 | 0.71 | 0.42 | 0.81 | 0.67 | 0.70 | 0.77 | 0.65 | 0.40 |
| 5 | 0.72 | 0.72 | 0.72 | 0.80 | 0.72 | 0.44 | 0.81 | 0.67 | 0.71 | 0.77 | 0.66 | 0.42 |
| 6 | 0.71 | 0.71 | 0.71 | 0.78 | 0.71 | 0.43 | 0.81 | 0.67 | 0.70 | 0.77 | 0.64 | 0.40 |
| 7 | 0.72 | 0.70 | 0.71 | 0.78 | 0.71 | 0.43 | 0.81 | 0.67 | 0.70 | 0.77 | 0.64 | 0.39 |
| 8 | 0.72 | 0.70 | 0.71 | 0.77 | 0.71 | 0.42 | 0.81 | 0.67 | 0.70 | 0.77 | 0.65 | 0.40 |
| **Ensemble** | 0.77 | 0.72 | 0.74 | .082 | 0.69 | 0.47 |  |  |  |  |  |  |

**Table S12:** 10-fold Cross-validation and test sensitivity, specificity, accuracy, AUC, F1-score, and MCC for datasets 1 to 8 using CNN.

| **Datasets** | **Cross-validation** | | | | | | **Test dataset** | | | | | |
| --- | --- | --- | --- | --- | --- | --- | --- | --- | --- | --- | --- | --- |
|  | **SN** | **SP** | **ACC** | **AUC** | **F1** | **MCC** | **SN** | **SP** | **ACC** | **AUC** | **F1** | **MCC** |
| 1 | 0.72 | 0.7 | 0.7 | 0.78 | 0.71 | 0.41 | 0.72 | 0.66 | 0.68 | 0.76 | 0.63 | 0.37 |
| 2 | 0.66 | 0.7 | 0.68 | 0.76 | 0.67 | 0.37 | 0.65 | 0.69 | 0.67 | 0.75 | 0.59 | 0.33 |
| 3 | 0.68 | 0.67 | 0.68 | 0.75 | 0.67 | 0.37 | 0.66 | 0.65 | 0.66 | 0.74 | 0.59 | 0.31 |
| 4 | 0.71 | 0.65 | 0.68 | 0.74 | 0.69 | 0.37 | 0.72 | 0.61 | 0.65 | 0.73 | 0.61 | 0.32 |
| 5 | 0.7 | 0.69 | 0.7 | 0.77 | 0.69 | 0.4 | 0.71 | 0.65 | 0.67 | 0.76 | 0.62 | 0.36 |
| 6 | 0.68 | 0.7 | 0.69 | 0.76 | 0.69 | 0.39 | 0.66 | 0.69 | 0.68 | 0.76 | 0.62 | 0.35 |
| 7 | 0.74 | 0.67 | 0.71 | 0.78 | 0.71 | 0.42 | 0.72 | 0.64 | 0.67 | 0.75 | 0.63 | 0.36 |
| 8 | 0.76 | 0.65 | 0.7 | 0.78 | 0.72 | 0.41 | 0.75 | 0.61 | 0.66 | 0.74 | 0.63 | 0.35 |
| **Ensemble** | 0.65 | 0.74 | 0.71 | .081 | 0.68 | 0.44 |  |  |  |  |  |  |

**Table S13:** 10-fold Cross-Validation and Test Sensitivity, Specificity, Accuracy, AUC, F1-score, and MCC for Datasets 1 to 8 using AAC.

| **Datasets** | **Cross-validation** | | | | | | **Test dataset** | | | | | |
| --- | --- | --- | --- | --- | --- | --- | --- | --- | --- | --- | --- | --- |
|  | **SN** | **SP** | **ACC** | **AUC** | **F1** | **MCC** | **SN** | **SP** | **ACC** | **AUC** | **F1** | **MCC** |
| 1 | 0.62 | 0.49 | 0.56 | 0.59 | 0.58 | 0.11 | 0.66 | 0.5 | 0.56 | 0.61 | 0.54 | 0.16 |
| 2 | 0.62 | 0.54 | 0.58 | 0.61 | 0.59 | 0.16 | 0.64 | 0.48 | 0.54 | 0.59 | 0.52 | 0.12 |
| 3 | 0.61 | 0.55 | 0.58 | 0.6 | 0.59 | 0.15 | 0.59 | 0.5 | 0.53 | 0.58 | 0.49 | 0.09 |
| 4 | 0.64 | 0.51 | 0.58 | 0.6 | 0.6 | 0.16 | 0.65 | 0.48 | 0.55 | 0.6 | 0.52 | 0.13 |
| 5 | 0.66 | 0.55 | 0.6 | 0.63 | 0.62 | 0.2 | 0.7 | 0.48 | 0.56 | 0.61 | 0.55 | 0.17 |
| 6 | 0.62 | 0.48 | 0.56 | 0.58 | 0.58 | 0.11 | 0.65 | 0.49 | 0.55 | 0.6 | 0.52 | 0.14 |
| 7 | 0.64 | 0.52 | 0.58 | 0.61 | 0.6 | 0.16 | 0.64 | 0.5 | 0.55 | 0.6 | 0.52 | 0.14 |
| 8 | 0.66 | 0.50 | 0.58 | 0.61 | 0.61 | 0.17 | 0.7 | 0.46 | 0.55 | 0.61 | 0.54 | 0.16 |
| **Ensemble** | 0.73 | 0.62 | 0.67 | 0.71 | 0.63 | 0.35 |  |  |  |  |  |  |

**Table S14:** 10-fold Cross-Validation and Test Sensitivity, Specificity, Accuracy, AUC, F1-score, and MCC for Datasets 1 to 8 using DPC.

| **Datasets** | **Cross-validation** | | | | | | **Test dataset** | | | | | |
| --- | --- | --- | --- | --- | --- | --- | --- | --- | --- | --- | --- | --- |
|  | **SN** | **SP** | **ACC** | **AUC** | **F1** | **MCC** | **SN** | **SP** | **ACC** | **AUC** | **F1** | **MCC** |
| 1 | 0.59 | 0.61 | 0.6 | 0.65 | 0.6 | 0.2 | 0.56 | 0.59 | 0.58 | 0.61 | 0.5 | 0.15 |
| 2 | 0.58 | 0.61 | 0.59 | 0.65 | 0.59 | 0.19 | 0.6 | 0.59 | 0.59 | 0.63 | 0.53 | 0.18 |
| 3 | 0.55 | 0.61 | 0.58 | 0.63 | 0.56 | 0.15 | 0.58 | 0.62 | 0.61 | 0.63 | 0.53 | 0.19 |
| 4 | 0.57 | 0.62 | 0.59 | 0.63 | 0.58 | 0.18 | 0.56 | 0.63 | 0.6 | 0.62 | 0.52 | 0.18 |
| 5 | 0.63 | 0.62 | 0.63 | 0.67 | 0.62 | 0.25 | 0.55 | 0.62 | 0.59 | 0.61 | 0.51 | 0.16 |
| 6 | 0.57 | 0.62 | 0.6 | 0.64 | 0.58 | 0.2 | 0.54 | 0.62 | 0.59 | 0.61 | 0.5 | 0.15 |
| 7 | 0.56 | 0.63 | 0.59 | 0.63 | 0.58 | 0.19 | 0.58 | 0.58 | 0.58 | 0.61 | 0.51 | 0.15 |
| 8 | 0.56 | 0.60 | 0.58 | 0.62 | 0.57 | 0.16 | 0.58 | 0.61 | 0.60 | 0.61 | 0.52 | 0.18 |
| **Ensemble** | 0.65 | 0.64 | 0.64 | 0.66 | 0.58 | 0.27 |  |  |  |  |  |  |

**Table S15:** 10-fold Cross-Validation and Test Sensitivity, Specificity, Accuracy, AUC, F1-score, and MCC for Datasets 1 to 8 using TPC.

| **Datasets** | **Cross-validation** | | | | | | **Test dataset** | | | | | |
| --- | --- | --- | --- | --- | --- | --- | --- | --- | --- | --- | --- | --- |
|  | **SN** | **SP** | **ACC** | **AUC** | **F1** | **MCC** | **SN** | **SP** | **ACC** | **AUC** | **F1** | **MCC** |
| 1 | 0.59 | 0.63 | 0.61 | 0.65 | 0.6 | 0.22 | 0.57 | 0.64 | 0.61 | 0.64 | 0.53 | 0.20 |
| 2 | 0.56 | 0.65 | 0.61 | 0.65 | 0.59 | 0.21 | 0.61 | 0.63 | 0.62 | 0.65 | 0.55 | 0.23 |
| 3 | 0.55 | 0.59 | 0.57 | 0.61 | 0.56 | 0.14 | 0.53 | 0.62 | 0.59 | 0.6 | 0.49 | 0.15 |
| 4 | 0.57 | 0.62 | 0.6 | 0.63 | 0.59 | 0.19 | 0.52 | 0.59 | 0.56 | 0.58 | 0.47 | 0.11 |
| 5 | 0.58 | 0.63 | 0.61 | 0.64 | 0.59 | 0.21 | 0.56 | 0.63 | 0.61 | 0.63 | 0.52 | 0.19 |
| 6 | 0.58 | 0.62 | 0.6 | 0.62 | 0.59 | 0.2 | 0.56 | 0.63 | 0.61 | 0.63 | 0.52 | 0.19 |
| 7 | 0.59 | 0.65 | 0.62 | 0.64 | 0.61 | 0.24 | 0.58 | 0.61 | 0.6 | 0.63 | 0.52 | 0.19 |
| 8 | 0.59 | 0.64 | 0.61 | 0.64 | 0.6 | 0.22 | 0.62 | 0.61 | 0.62 | 0.65 | 0.55 | 0.23 |
| **Ensemble** | 0.55 | 0.69 | 0.64 | 0.65 | 0.53 | 0.24 |  |  |  |  |  |  |

**Table S16:** 10-fold Cross-Validation and Test Sensitivity, Specificity, Accuracy, AUC, F1-score, and MCC for Datasets 1 to 8 using CTD.

| **Datasets** | **Cross-validation** | | | | | | **Test dataset** | | | | | |
| --- | --- | --- | --- | --- | --- | --- | --- | --- | --- | --- | --- | --- |
|  | **SN** | **SP** | **ACC** | **AUC** | **F1** | **MCC** | **SN** | **SP** | **ACC** | **AUC** | **F1** | **MCC** |
| 1 | 0.74 | 0.28 | 0.52 | 0.5 | 0.6 | 0.02 | 0.69 | 0.31 | 0.45 | 0.51 | 0.48 | 0.01 |
| 2 | 0.7 | 0.34 | 0.53 | 0.49 | 0.59 | 0.04 | 0.7 | 0.34 | 0.48 | 0.48 | 0.5 | 0.04 |
| 3 | 0.71 | 0.35 | 0.53 | 0.48 | 0.59 | 0.06 | 0.7 | 0.33 | 0.47 | 0.49 | 0.49 | 0.04 |
| 4 | 0.73 | 0.32 | 0.53 | 0.48 | 0.6 | 0.06 | 0.69 | 0.32 | 0.46 | 0.49 | 0.48 | 0.01 |
| 5 | 0.72 | 0.32 | 0.53 | 0.52 | 0.59 | 0.05 | 0.7 | 0.32 | 0.47 | 0.51 | 0.49 | 0.03 |
| 6 | 0.68 | 0.34 | 0.52 | 0.52 | 0.56 | 0.03 | 0.67 | 0.34 | 0.47 | 0.51 | 0.47 | 0.02 |
| 7 | 0.73 | 0.35 | 0.55 | 0.52 | 0.6 | 0.08 | 0.69 | 0.33 | 0.47 | 0.51 | 0.48 | 0.03 |
| 8 | 0.72 | 0.34 | 0.54 | 0.48 | 0.6 | 0.08 | 0.7 | 0.32 | 0.47 | 0.49 | 0.5 | 0.03 |
| **Ensemble** | 0.52 | 0.63 | 0.59 | 0.42 | 0.49 | 0.15 |  |  |  |  |  |  |

**Table S17:** 10-fold Cross-Validation and Test Sensitivity, Specificity, Accuracy, AUC, F1-score, and MCC for Datasets 1 to 8 using 1H encodings.

| **Datasets** | **Cross-validation** | | | | | | **Test dataset** | | | | | |
| --- | --- | --- | --- | --- | --- | --- | --- | --- | --- | --- | --- | --- |
|  | **SN** | **SP** | **ACC** | **AUC** | **F1** | **MCC** | **SN** | **SP** | **ACC** | **AUC** | **F1** | **MCC** |
| 1 | 0.62 | 0.59 | 0.61 | 0.63 | 0.6 | 0.22 | 0.63 | 0.54 | 0.58 | 0.62 | 0.53 | 0.18 |
| 2 | 0.69 | 0.57 | 0.63 | 0.64 | 0.65 | 0.26 | 0.68 | 0.55 | 0.6 | 0.64 | 0.56 | 0.22 |
| 3 | 0.64 | 0.55 | 0.6 | 0.59 | 0.60 | 0.20 | 0.63 | 0.54 | 0.57 | 0.6 | 0.52 | 0.16 |
| 4 | 0.66 | 0.56 | 0.61 | 0.62 | 0.63 | 0.22 | 0.61 | 0.53 | 0.56 | 0.6 | 0.51 | 0.14 |
| 5 | 0.71 | 0.53 | 0.62 | 0.64 | 0.65 | 0.25 | 0.67 | 0.48 | 0.55 | 0.62 | 0.53 | 0.15 |
| 6 | 0.64 | 0.58 | 0.62 | 0.61 | 0.62 | 0.23 | 0.58 | 0.59 | 0.59 | 0.61 | 0.51 | 0.17 |
| 7 | 0.68 | 0.57 | 0.62 | 0.61 | 0.64 | 0.25 | 0.61 | 0.53 | 0.56 | 0.6 | 0.51 | 0.14 |
| 8 | 0.74 | 0.49 | 0.62 | 0.62 | 0.66 | 0.25 | 0.71 | 0.43 | 0.54 | 0.61 | 0.54 | 0.14 |
| **Ensemble** | 0.65 | 0.64 | 0.64 | 0.64 | 0.58 | 0.28 |  |  |  |  |  |  |

**Table S18:** 10-fold Cross-Validation and Test Sensitivity, Specificity, Accuracy, AUC, F1-score, and MCC for Datasets 1 to 8 using Prot5 encodings.

| **Datasets** | **Cross-validation** | | | | | | **Test dataset** | | | | | |
| --- | --- | --- | --- | --- | --- | --- | --- | --- | --- | --- | --- | --- |
|  | **SN** | **SP** | **ACC** | **AUC** | **F1** | **MCC** | **SN** | **SP** | **ACC** | **AUC** | **F1** | **MCC** |
| 1 | 0.41 | 0.65 | 0.55 | 0.54 | 0.39 | 0.1 | 0.40 | 0.63 | 0.54 | 0.52 | 0.32 | 0.04 |
| 2 | 0.41 | 0.64 | 0.54 | 0.54 | 0.39 | 0.08 | 0.41 | 0.63 | 0.54 | 0.52 | 0.33 | 0.05 |
| 3 | 0.58 | 0.44 | 0.53 | 0.52 | 0.47 | 0.04 | 0.57 | 0.45 | 0.50 | 0.52 | 0.39 | 0.03 |
| 4 | 0.33 | 0.74 | 0.55 | 0.54 | 0.35 | 0.11 | 0.32 | 0.72 | 0.56 | 0.52 | 0.29 | 0.05 |
| 5 | 0.33 | 0.75 | 0.55 | 0.54 | 0.35 | 0.11 | 0.32 | 0.72 | 0.56 | 0.52 | 0.29 | 0.06 |
| 6 | 0.41 | 0.63 | 0.54 | 0.53 | 0.38 | 0.06 | 0.4 | 0.63 | 0.54 | 0.52 | 0.33 | 0.05 |
| 7 | 0.40 | 0.64 | 0.54 | 0.52 | 0.37 | 0.06 | 0.39 | 0.64 | 0.54 | 0.52 | 0.3 | 0.04 |
| 8 | 0.41 | 0.64 | 0.54 | 0.53 | 0.39 | 0.08 | 0.40 | 0.63 | 0.54 | 0.51 | 0.33 | 0.04 |
| **Ensemble** | 0.15 | 0.90 | 0.61 | 0.53 | 0.23 | 0.07 |  |  |  |  |  |  |

**Table S19:** 10-fold Cross-Validation and Test Sensitivity, Specificity, Accuracy, AUC, F1-score, and MCC for Datasets 1 to 8 using Unirep encodings.

| **Datasets** | **Cross-validation** | | | | | | **Test dataset** | | | | | |
| --- | --- | --- | --- | --- | --- | --- | --- | --- | --- | --- | --- | --- |
|  | **SN** | **SP** | **ACC** | **AUC** | **F1** | **MCC** | **SN** | **SP** | **ACC** | **AUC** | **F1** | **MCC** |
| 1 | 0.50 | 0.79 | 0.64 | 0.67 | 0.58 | 0.3 | 0.48 | 0.77 | 0.66 | 0.67 | 0.52 | 0.26 |
| 2 | 0.52 | 0.79 | 0.66 | 0.68 | 0.59 | 0.33 | 0.48 | 0.77 | 0.66 | 0.67 | 0.51 | 0.26 |
| 3 | 0.58 | 0.74 | 0.65 | 0.67 | 0.64 | 0.32 | 0.54 | 0.7 | 0.64 | 0.66 | 0.53 | 0.24 |
| 4 | 0.58 | 0.78 | 0.67 | 0.7 | 0.65 | 0.37 | 0.58 | 0.72 | 0.67 | 0.69 | 0.57 | 0.3 |
| 5 | 0.58 | 0.74 | 0.66 | 0.68 | 0.64 | 0.33 | 0.57 | 0.68 | 0.64 | 0.67 | 0.55 | 0.26 |
| 6 | 0.59 | 0.74 | 0.66 | 0.69 | 0.65 | 0.34 | 0.57 | 0.7 | 0.65 | 0.69 | 0.56 | 0.27 |
| 7 | 0.62 | 0.72 | 0.67 | 0.69 | 0.66 | 0.34 | 0.58 | 0.7 | 0.65 | 0.69 | 0.56 | 0.28 |
| 8 | 0.82 | 0.64 | 0.76 | 0.77 | 0.82 | 0.46 | 0.8 | 0.45 | 0.58 | 0.67 | 0.59 | 0.25 |
| **Ensemble** | 0.56 | 0.78 | 0.70 | 0.72 | 0.58 | 0.35 |  |  |  |  |  |  |
